# HIV-associated neurocognitive disorders: recent advances in pathogenesis, biomarkers, and treatment

**DOI:** 10.12688/f1000research.10651.1

**Published:** 2017-03-23

**Authors:** Antonia Carroll, Bruce Brew

**Affiliations:** 1 Department of Neurology, St Vincent’s Hospital, Level 4, Xavier Building, Victoria Street, Darlinghurst, Sydney, Australia; 2 Peter Duncan Neurosciences Unit, St Vincent’s Centre for Applied Medical Research, St Vincent’s Hospital, Sydney, Australia; 3 Department of HIV Medicine, St Vincent’s Hospital, Level 4, Xavier Building, Victoria Street, Darlinghurst, Sydney, Australia; 4 University of New South Wales, St. Vincent’s Clinical School, Delacy Building, Victoria Street, Darlinghurst, Sydney, Australia

**Keywords:** HIV-associated neurocognitive disorders, HAND, HIV, CNS, HAART

## Abstract

HIV-associated neurocognitive disorders (HAND) remain prevalent despite plasma viral suppression by antiretroviral agents. In fact, the prevalence of milder subtypes of cognitive impairment is increasing. Neuropsychologic testing remains the “gold standard” of diagnosis; however, this is time consuming and costly in a resource-poor environment. Recently developed screening tools, such as CogState and the revised HIV dementia scale, have very good sensitivity and specificity in the more severe stages of HAND. However, questions remain regarding the utility of, optimal population for, and insensitivity of tests in mild HAND.

Recognition of ongoing viral persistence and the inflammatory milieu in the central nervous system (CNS) has advanced our understanding of the pathogenesis of HAND and facilitated the development of biomarkers of CNS disease. The importance of the monocyte-macrophage lineage cell and the astrocyte as viral reservoirs, HIV viral proteins, self-perpetuating CNS inflammation, and CCR5 chemokine receptor neurotropism has been identified. Whilst biomarkers demonstrate monocyte activation, inflammation, and neuronal injury, they remain limited in their clinical utility. The improved understanding of pathogenic mechanisms has led to novel approaches to the treatment of HAND; however, despite these advances, the optimal management is still undefined.

## Introduction

In the era of highly active antiretroviral therapy (HAART) for human immunodeficiency virus (HIV), there has been a dramatic shift in neurologic sequelae of the disease. Opportunistic infections associated with severe immunodeficiency are now much less prevalent, but neurologic complications associated with chronic infection, treatments, immune reconstitution, and concurrent comorbidities are increasing. Despite HAART, HIV-associated neurocognitive disorders (HAND) remain a chronic issue, with significant effects on an individual’s ability to perform activities of daily living, quality of life, employment, medication adherence, and survival ^1– 5^.

HAND is a common disease with variable reported prevalence in different populations (20–69%) ^6– 14^. It is most commonly seen in advanced stages of HIV/AIDS; however, HAND can also occur in asymptomatic HIV infection ^12, 15– 18^. It is critically important, though, to recognize that HAND occurs in only *some* patients – the majority of patients who are virally suppressed on HAART will not develop HAND. HAND is grouped by the 2007 Frascati criteria into three categories of increasing severity of cognitive impairment: asymptomatic neurocognitive impairment (ANI), mild neurocognitive dementia (MND), and HIV-associated dementia (HAD) ^19^. The category is assigned on the basis of clinical features, especially those related to daily functioning, exclusion of alternative causes, and neuropsychological testing.

HAART has transformed HIV treatment, controlling plasma viremia and allowing the life expectancy of HIV-positive patients to approach that of age-matched controls ^20^. While HAART has reduced the incidence of all categories of moderate to severe dementia (HAD: 7% in 1989 to 1% in 2000) ^21^, there is a continued and increasing prevalence of milder subtypes of HAND – ANI and MND – despite sustained virologic control ^14, 15, 17, 22^. ANI now accounts for between 33 and 60% of all HAND ^12, 14, 17^. Thus, HAART has significantly impacted upon only the more severe forms of HAND, but one would expect these potent antiretrovirals (ARVs) to have a more profound effect on milder HAND. We have termed this curious situation the “therapeutic paradox”. The “natural history” of HAND in the context of viral suppression has also been clarified recently. Most patients who continue to be virally suppressed remain stable, but some improve and a small number deteriorate ^23^. This is best termed as the *activity* of HAND.

Despite the terminology, ANI is not without consequence, with negative impacts on quality of life, medication adherence, and employment ^1– 5^, and some studies show progression to MND/HAD ^14, 24^. As such, HAND remains an important ongoing area of clinical research in the context of increasing worldwide impact of HAND as HAART becomes more prevalent. This update summarizes recent advances in central nervous system (CNS) HIV disease, including screening strategies for HAND, its pathogenesis, biomarkers for HAND, and its treatment.

## Screening

The uncertainty in the natural history of HAND raises the question of whether screening for HAND is beneficial, with some groups suggesting that screening could lead to unnecessary, costly, invasive procedures in a climate of limited clinical resources. For screening to be beneficial, tests with adequate sensitivity and specificity are required, as well as management that alters the course of disease, preventing progression from early stages to severe dementia. While progress has recently been made with regard to these requirements (e.g. 25– 27), at the present time neither of these criteria are definitively met.

Multiple HIV-specific factors have been associated with cognitive decline. Longer duration of HIV, nadir CD4 ^+^ T cell count, ongoing viremia, and replication in the CNS and periphery are all associated with cognitive decline ^9, 14, 28– 31^. In particular, the risk of HAND increases as CD4 ^+^ counts decline below 350 cells/µL and with higher plasma HIV viral loads ^30^. These HIV-specific factors are potentially preventable or treatable with alterations to medication regimes, suggesting that early intervention may be able to reduce the risk of HAND; however, this hypothesis is yet to be confirmed (see the section titled “Treatment”). As a result, we feel that improved screening methods to identify early those developing HAND and, in particular, high-risk individuals remains important for our ability to combat HAND in the future.

Screening tests capturing mild cognitive impairments are currently being investigated. A newly designed computerized battery, CogState, assessing five relevant cognitive domains has recently been assessed in Australia in HIV-positive individuals relative to age- and education-matched controls ^26^. Comparison with neuropsychological testing resulted in a sensitivity of 73% and specificity of 82%, with a correct classification rate of 79% ^26^. In the MND/HAD subgroup, the sensitivity and specificity improved dramatically to 100% and 98%, respectively ^26^. This screening method is suitable for application by non-specialists and may assist with detecting patients requiring more in-depth neuropsychological testing, hence guiding limited resources in this setting.

While this test does not improve screening for the ANI category of patients, these individuals could be monitored with longitudinal re-screening whilst controlling and treating alternate risk factors for cognitive decline ^26^. Further studies will be required to assess the validity of this screening test in longitudinal studies. Thus far, longitudinal decline, as measured with repeat assessments of the HIV dementia scale (HDS), was reliable in the retest setting; however, similar to CogState, this technique was also able to robustly detect only moderate to severe cognitive decline ^24^.

Despite the limitations defined above, the guidelines currently suggest that screening for cognitive impairment should be performed in HIV-seropositive individuals; however, no consensus has been reached regarding the time point or screening tools that should be used ^32– 35^. Screening has not yet been universally adopted by practicing clinicians. Nonetheless, at present, it would seem reasonable for screening to target those individuals at risk of HAND rather than the general HIV population. Such risk factors have been discussed in the second paragraph of this section. It is not clear yet whether some or all of these should be used as “red flags” for screening, but at least they serve as a starting point.

## Pathogenesis and the evolution of biomarkers

The therapeutic paradox has called into question our understanding of the pathophysiology of HAND. Treatments targeting these mechanisms may be the key to effective treatment in the future. While an in-depth review of the pathophysiology of HIV in the CNS is beyond the scope of this review, a few key advances have been identified in recent years, including the importance of monocyte–macrophage and astrocyte viral reservoirs, chronic inflammation in the CNS, HIV viral proteins, and CCR5 chemokine receptor neurotropism.

### The brain as a viral reservoir

The concept of quiescent viral reservoirs, systemically or in the CNS, has emerged as a barrier to HIV eradication in the post-HAART era ^36^. Monocytes and macrophages, in both the CNS and the periphery, express co-receptors necessary for HIV infection, are infected early in the course of disease, are long-living, do not die as a consequence of HIV infection or immune surveillance, and hence can become viral reservoirs for HIV ^37– 41^. Furthermore, ARV entry into the brain can be variable because of the blood–brain barrier, and ARV efficacy as well as the ability of agents to reverse latency in brain cells such as microglia and particularly astrocytes is at best limited ^42^. These factors point to the brain as a sanctuary site, further reinforcing the brain as a likely viral reservoir. Probable latent brain infection in macrophages, astrocytes, and other glial cells has been demonstrated in multiple neuropathologic and *in vitro* studies ^43– 50^. The issue remains controversial, though, as some consider that the demonstration of such virus reflects only the phagocytic function of those cells. Further, as yet, the virus in such cells has not been demonstrated to be replication competent, with the exception of macrophages, though the study used animal models ^51^. Nonetheless, one study found that after 10 years of suppressive antiretroviral therapy (ART) in the plasma, low levels of HIV RNA could still be detected in the cerebrospinal fluid (CSF) – that is, viral escape – with associated evidence of immune activation, thereby emphasizing the brain as both a sanctuary site and a viral reservoir ^52^.

Further support for the brain as a viral reservoir comes from a recent study of simian immunodeficiency virus (SIV)-infected macaques that were continuously virally suppressed with CNS-penetrant HAART. When latency-reversing agents were administered, there was reactivation of virus in the macaque brain ^53^. This resulted in increases in CSF viral load, 10 times higher than plasma, and increased CNS immune activation and neuronal damage markers, likely contributing to a harmful CNS inflammatory response ^53^. The most prevalent SIV genotype in the CSF was novel compared with peripheral genotypes, suggesting that distinct genomes that persisted in the CNS compartment were amplified despite long-term peripheral viral suppression ^53^. Similarly, in humans, it is relatively common to find different genotypes of HIV in the CNS and periphery, again demonstrating compartmentalization in the CNS and its role as a reservoir ^54, 55^.

### Monocyte populations and biomarkers

Direct evidence of infected monocytes entering the brain has not been demonstrated in humans; however, animal models have demonstrated peripheral monocyte trafficking to the CNS in SIV infection ^56^. HIV viral load and proviral HIV DNA in plasma mononuclear cells have been linked to disease progression, AIDS development, and HAND in HAART-naïve advanced infection and proviral DNA in virally suppressed individuals ^57– 59^. Interestingly, a recent study identified that levels of HIV DNA in peripheral mononuclear cells were not associated with HAND in virally suppressed HIV-positive individuals on stable HAART regimens. Instead, an increase in monocyte HIV-positive reservoir size was associated with progression of HAND, in particular in the HAD subgroup ^60^. These papers raise a crucial issue in HAND pathogenesis: at what point do systemic factors become less important compared to brain-related factors? In other words, when does systemic HIV disease “metastasize” to the brain, at that point making it a truly brain-autonomous problem no longer driven by systemic disease?

In HIV, the phenotypic subpopulations of monocytes are altered, resulting in increased circulating CD16 ^+^ monocytes instead of the usually prevalent CD14 ^+^CD16 ^–^ populations found in normal controls ^61, 62^. CD16 ^+^ cells can be infected by HIV, in part due to differences in host restriction factor expression (APOBEC isoforms and SAMDH1) allowing HIV replication ^63– 68^. The proportion of CD16 ^+^ monocytes in the total population is associated with uncontrolled viremia (>400 copies/mL), reduced by adherence to ART, and linked to disease progression ^61, 62, 69, 70^. Increased proportions and absolute numbers of CD16 ^+^ monocytes are more strongly associated with HAND than with the CSF viral load or proportion of HIV-infected cells in the CNS; hence, measurement of CD16 ^+^ populations, CD14 ^+^/CD16 ^+^ ratio, and high HIV DNA levels in CD16 ^+^ cells may be used as biomarkers of disease and correlate with neurocognitive dysfunction ^71– 75^.

Alternate monocyte biomarkers have also been identified. CD163 is a hemoglobin–haptoglobin scavenger receptor found exclusively on monocytes and macrophages ^76– 80^. When monocytes are activated by lipopolysaccharide, Fc *γ* receptors crosslink or oxidative stress occurs, CD163 is shed as soluble CD163 (sCD163) to reduce inflammatory cell activation and cytokine release ^77, 78, 81– 83^. Expression of CD163 is higher on CD16 ^+^ monocytes ^84^. Plasma sCD163 is elevated in individuals with HIV duration greater than one year, and HAART therapy reduces sCD163 commensurate with HIV RNA levels; however, sCD163 levels remain significantly higher than in controls, suggesting ongoing low-level monocyte activation ^83^. Follow-up clinical studies demonstrate that higher levels of sCD163 occur in HAND populations, in particular the MND/HAD groups, compared with those who were cognitively unimpaired ^85^. These results suggest that sCD163 is a novel marker of macrophage-mediated disease activity in HIV, systemically and in the CNS. However, it is not specific for HIV, nor does it distinguish the duration of infection or HAND subtype.

Another marker of monocyte activation, sCD14, a soluble monocyte lipopolysaccharide receptor, is similar to sCD163 in that levels are higher in early and chronic HIV infection than in HIV-negative controls ^83^. However, sCD14 levels were not reduced by treatment with HAART and were significantly increased after treatment with HAART in early HIV ^83^. Plasma and CSF sCD163 correlates with sCD14 ^83, 85^. However, despite this, sCD14 has had variable associations with HAND severity ^85, 86^. Alternate monocyte biomarkers, such as CSF CCL2, correlate with HAND severity and risk of progression as well as markers of inflammation ^87, 88^. The comparative clinical utility of these biomarkers requires further study.

### Chronic inflammation and HIV proteins

The inflammatory milieu, as a potential contributor to HAND, has become an interesting area of research. It is increasingly recognized that low-level latent infection in HIV and its comorbidities (e.g. metabolic syndrome, drug use, and hepatitis C) as well as aging can lead to chronic low-grade immune activation and inflammation ^89, 90^. Sustained CNS inflammation occurring during HAART may be due to latent and low-level infection in the CSF meningeal compartment and possibly brain (viral escape), priming of microglia and macrophages due to circulating products translocated from the gut, altered neuronal and synaptic function, contributions from the metabolic syndrome, and possible ART-related toxicity.

The macrophage and microglia biomarker of immune activation, neopterin, is elevated in most HIV seropositive individuals, with levels declining after commencement of HAART. Low viral persistence in the CNS, as measured by CSF HIV RNA, is significantly correlated with CSF neopterin elevation ^91^. Neopterin correlates with HAND severity in ART-naïve individuals and predicts the development of HAND; however, its utility in the HAART era is not clear ^92– 94^.

Inflammatory cytokine secretion by infected monocytes causes a cascade, with compounding effects due to positive feedback on gene transcription ^95^. This chronic inflammation is probably exacerbated by HIV component proteins expressed in infected cells (especially monocytes), such as Vpr, Tat, Nef, and gp120, which further activate inflammatory pathways, leading directly to neural toxicity ^96– 99^. However, chronic inflammation in latent HIV infection with suppressed HIV RNA may be caused by deregulation of inflammatory cascades by transcriptional regulators, such as BCL11B, with resultant silencing of integrated virus ^43^. Conversely, in HIV-encephalitis (HIVE), reduced transcriptional regulation leads to re-emergence of HIV RNA in CSF and brain ^43^. This suggests that HAND with viral suppression may be driven by viral components, such as Tat and Nef, whereas HAND without viral suppression may be fuelled by whole virus, in particular envelope proteins. This is an attractive potential explanation for HAND in the context of viral suppression, as current HAART does not target the immediate post-integration transcription stage of the HIV replication cycle, allowing the continued production of Tat, Nef, and Vpr.

Tat and Nef have been found to affect endothelial activation and dysfunction, leading to cardiovascular disease ^100^. Tat transcriptionally deregulates genes and induces cytokine activation of IL-17 pathways, leading to a proinflammatory state with direct neurotoxicity ^101^. In addition, it is soluble, able to exit the infected cell, and can affect neighboring cells ^101^. Thus, measurement of Tat is a plausible biomarker for neural damage in HAND. However, CSF Tat does not correlate directly with HAND severity, possibly because it is only intermittently produced, or there may be difficulties with the lower limit of detection in current assays ^102^. In the future, antisense oligonucleotides and monoclonal antibodies could be utilized to enhance the efficacy of HAART ^103^.

### CCR5 chemokine receptor neurotropism

Viral envelope glycoproteins can fuse with only those cells expressing both CD4 and an HIV co-receptor, of which CCR5 has been identified as the most important for microglia and CNS macrophages ^104, 105^. CCR5 receptors are upregulated on activated CD4 ^+^ and CD8 ^+^ T cells, enhancing antigen-presenting cell interaction, T cell trafficking into tissues, and cytokine production ^106^. In response to infection or inflammation, monocytes, microglia, astrocytes, and neurons express CCR5 ligands, which increase the migration of CCR5 ^+^ T cells into the CNS ^106^. Locally, these effector T cells secrete CCR5 ligands, which amplify the immune response ^106^. A subset of CCR5-tropic viruses have been found to be highly fusogenic, requiring lower expression levels of CCR5 and CD4 to infect cells, leading to greater apoptosis ^107^. There is now good evidence that the vast majority of HIV strains in the brain are CCR5 tropic ^108^. Given the intrinsic role of CCR5 in CNS disease, it is suggested that CCR5 antagonists, such as maraviroc, may be able to reduce the migration and then activation of effector CD8 ^+^ T cells in the CNS and hence reduce the neurocognitive sequelae of HIV ^106^. This is now being assessed (see the section titled “Treatment”).

### Biomarkers of HIV replication

While quantification of CSF HIV DNA as a biomarker of HAND is both impracticable and insensitive ^109^, measurement of HIV RNA expression in CSF is more robust. In the pre-HAART era and ARV-naïve population, HIV RNA in blood is independently associated with cognitive impairment ^110^, high levels have been associated with HAD in individuals with advanced immunosuppression, and a decrease in HIV RNA levels has been correlated with improvements in cognitive measures ^111– 115^. While these factors suggest that HIV RNA level can be a useful biomarker in HAND, it is by no means a perfect marker; its utility in the HAART era is less well established. CSF HIV RNA levels may not accurately reflect HIV replication in brain parenchyma, though single copy assay techniques hold some promise.

### Biomarkers of neural injury

Biomarkers of neural damage would be expected to correlate most closely with HAND. CSF neurofilament light chain (NFL), a component of myelinated axons, has recently been identified as a sensitive biomarker of neuronal injury in multiple neurodegenerative diseases ^116– 118^. In HIV, CSF NFL is increased in patients with and without HAND, with levels higher in HAND and increasing as HIV disease advances, suggesting early neuronal injury ^116^. CSF NFL concentrations appear to predict the development of HAD, with levels increasing 1–2 years before symptoms develop ^119^. However, NFL levels are not sensitive in the detection of mild disease, in particular when virally suppressed ^118^. Newer ultrasensitive plasma assays for NFL are highly correlated with CSF NFL concentrations ^117^. NFL levels in plasma and CSF increase with reducing CD4 counts and increasing plasma RNA viral loads in asymptomatic individuals, correlate with HAND severity, reaching the highest values in HAD, and are reduced by HAART ^117, 118, 120^. NFL is significantly correlated with sCD163 and sCD14, suggesting that CNS monocyte activation is directly linked with neuronal injury in HAND ^118^. Plasma NFL, although not specific, may be an emerging sensitive, non-invasive assay for HAND, especially as the sensitivity of assays increases.

Other biomarkers of neural damage such as tau and CSF/plasma albumin ratios, reflecting blood–brain barrier integrity, correlate with HAD and in some studies CSF neopterin ^121– 123^. These, however, are significantly reduced in the setting of HAART and hence are less useful in the clinical setting of HAND with viral suppression ^121^. In addition, non-invasive quantitative MRI techniques can reflect neuroinflammation and neuronal injury. A recent study demonstrated early reductions in parenchymal volume, enlargement of the brainstem and third ventricle, diffusion alterations in the caudate, altered white matter integrity in the corpus callosum, and brain metabolite changes reflecting neural injury within 100 days of seroconversion ^124^.

An additional emerging area of interest is microRNAs, the non-coding RNA molecules which regulate gene expression. Specific subtypes miR125b and 146a have a role in microglial infection and cell death ^125^. In a small study of 10 patients, nine with HAND, these microRNAs correlated with HIVE; however, the interaction with ART and viral suppression has yet to be elucidated ^125^.

## Treatment

Recent studies in HIV treatment have attempted to answer three main questions: firstly, is there a role for neuroHAART to improve CNS penetration and concentrations of ARVs? Secondly, is there a role for HAART intensification for HAND in the context of viral suppression? And, thirdly, can HAND be reduced by earlier HAART initiation?

### CNS penetration by HAART

The CNS penetration effectiveness (CPE) score was proposed in order to rank drug penetration and efficacy in the CNS ^126^. The CPE score has been supported in most studies, where treatment regimens with a higher CPE score generally have lower rates of HIV RNA detectible in CSF ^127, 128^. However, the issue of the utility of the CPE score remains controversial. Indeed, the CPE score is limited by its categorical scoring, unclear weighting of each criterion (pharmacokinetic, chemical properties, etc.), and lack of consideration of toxic effects or drug interactions. Furthermore, the CPE score relies on CSF drug concentrations, which may not reflect the brain parenchymal pharmacokinetics, and efficacy in glial cells is not considered, primarily due to limited data availability.

To assess this latter point, recent studies assessing the efficacy of current CNS-penetrating ARVs in astrocytes and macrophage-lineage cells have been performed using *in vitro* models of infection ^42, 129^. All of the agents tested could inhibit HIV infection in macrophages with concentrations typically found in CSF ^42^. Similar results were found in astrocytes, except for lamivudine, stavudine, and zidovudine, which required between 12- and 187-fold greater concentrations than are achievable in the CSF ^42^. These results indicate that these drugs may not adequately treat astrocyte reservoirs and suggest that these agents should not be utilized in CNS-penetrating (NeuroHAART) regimens.

The interaction between improved NeuroHAART, commonly defined by CPE score, and clinical efficacy has not been clearly elucidated. A recent systematic review found only six methodologically sound and two sufficiently powered studies that attempted to answer this question ^130^. Utilizing these more rigorous studies, it was concluded that a regimen with higher CNS penetration probably improved both neurocognitive function and HIV CSF RNA concentrations ^130^. Subsequently, a multisite randomized controlled trial has been performed which showed no benefit for a NeuroHAART regimen ^131^. However, there was an imbalance at entry for CD4 nadir and hepatitis C status, factors which are known to influence neurocognition. Also, it is known that HAND patients may continue to improve on a stable HAART regimen for approximately 1 year from regimen commencement; the trial requirement for stability for only 8 weeks may have been a further confounding factor. As a result, there remain no definitive HAART guidelines for HAND. Further investigations with randomized controlled trials addressing the latter shortcomings are needed ^130^.

### HAART intensification for HAND in the context of viral suppression

Recent advances suggest that CCR5 receptor interactions with CNS reservoir cells are intrinsically linked with CNS HIV and HAND. Maraviroc, a CCR5 receptor antagonist, is a target for HAART intensification, as it has adequate CSF penetration, has low rates of resistance, inhibits CNS viral replication, including in monocyte/macrophage cells, and has anti-inflammatory properties in the CNS ^132– 137^. Maraviroc acts by blocking HIV entry into cells, hence restricting virion replication and indirectly inhibiting immediate post-integration viral protein production, which probably drives the CNS inflammatory milieu ^96– 99, 138^. A recent prospective, open-label pilot randomized controlled trial in individuals with viral suppression and stable ART for 12 months found maraviroc-intensified HAART improved global neurocognitive performance at both 6 and 12 months without significant side effects ^27^. In the future, large randomized controlled studies will be necessary to confirm these findings; however, this suggests that maraviroc intensification of HAART could be an option for the clinical management of HAND occurring in the context of viral suppression.

### Early initiation of HAART

Lower HIV DNA levels in monocytes are associated with HAART initiation within the first year of infection, suggesting that the early commencement of HAART may improve outcomes in HAND ^52, 139^. However, the impact of immediate versus deterred HAART on neurocognitive performance in ARV-naïve patients without profound immunosuppression is unclear. The recent INSIGHT START neurology substudy aimed to assess this ^140, 141^. Individuals were randomized to commence HAART either at baseline (CD4 >500 cells/μL) or when CD4 ^+^ cells were below 350 cells/μL ^140^. While serious AIDS and non-AIDS events were reduced in the immediate treatment arm, without increased adverse events, the neurocognitive substudy did not demonstrate similar improvements on neuropsychological tests ^140, 141^. However, less than 50% of the study population reached 4 years of follow-up, the potentially neurotoxic ARV, efavirenz, was commonly used ^142^, and low rates of HAND were found owing to short disease duration and high CD4 ^+^ counts. As a result, further studies with longer duration of follow-up and adequate power are required to assess the feasibility of CNS protection with early ART initiation.

### Future treatment directions

Current studies are investigating intranasal insulin, IL-1 antagonists, paroxetine, and complement inhibitors as well as limiting the replication of viral transcripts by Tat monoclonal antibodies and antisense oligonucleotides.

## Conclusion

Recently, significant advances have been made in our understanding of HAND, facilitating further development of disease biomarkers and new treatments. Three key principles have become evident: selectivity (only some patients develop HAND), activity (HAND may fluctuate over months with some patients improving, a minority deteriorating, and a majority remaining stable), and a dynamic systemic–brain interface (some HAND patients appear to have the disease driven by systemic factors, while brain-related factors are important in others). Recognition of each of these will be needed to address the following questions:

1. In the context of earlier ART institution, adherence, and longstanding viral suppression, will the incidence of HAND remain as high? If so, does this reflect the effect of the viral reservoir and sanctuary, the self-perpetuating inflammatory cascade, or both?
2. Can screening tests and biomarkers be optimized to assess for early stages of HAND and can treatments prevent the progression to more severe subtypes?
3. Can early sensitive plasma, CSF, and imaging biomarkers of CNS impairment be used to improve therapeutic clinical trials? Can a combination of methods provide a more holistic concept of HAND pathogenesis? Can these methods be applied to patient care in the clinic?
4. Can the early institution of ARVs limit the development of the CNS reservoirs and prevent the inflammatory milieu from developing?
5. Can improved CNS penetration and efficacy, in particular with the use of maraviroc-enriched regimes, whilst maintaining systemic viral suppression, reduce the incidence of HAND?
6. Can novel cellular and molecular therapies, targeting recently understood pathogenic pathways, improve therapeutic efficacy?

Despite the inherent difficulties, large-scale, prospective, randomized trials with longitudinal follow-up, strict and consistent definitions of HAND, and adequate evaluation of comorbid conditions remain the best way to further our knowledge of HAND. This will allow us to evaluate biomarkers in screening and monitoring roles and adequately examine the effect of novel treatment regimens on cognitive function.
